# The National Pension Fund

**Published:** 1887-11-05

**Authors:** 


### THE NATIONAL PENSION FUND.

To meet the wishes of many interested in this Fund who reside away from London it has been determined to hold an afternoon conference at the Society of Arts during the first fortnight in December on a day yet to be fixed. Meanwhile, any who wish points to be cleared up, or who have found a difficulty in understanding the scheme, will facilitate its successful initiation by communicating them in writing to Mr. Burdett, The Lodge, Porchester Square, W.

**Leicester Institution of Trained Nurses.** The committee of this Institution is thanked for their offer to submit to the nurses in their employ the plan for establishing a National Pension Fund, when it is approved, and to use their influence in inducing their staff to enrol their names as members, providing they can approve the principle upon which the Fund is based. The Leicester Institution employs a staff of thirty trained nurses and four probationers.

**What Constitutes Eligibility.** The Fund is intended for trained nurses, that is, certificated nurses, but it will rest with the committee appointed to administer the Fund to decide on each application on its merits, providing it is made by a trained nurse. It appears that some Guardians have raised the nurses' wages in workhouse infirmaries from £20 to £26 per annum, with rations, uniform, and washing, to enable each employé to subscribe to a Pension Fund. Such cases are referred to by the matron of the Bethnal Green Infirmary, who may encourage her nurses to register their names as desirous of joining the Fund, and so enable the case of each of her staff to be considered without prejudice.

**A Dowry on Marriage.** The nurse matron of the Chesham Cottage Hospital is thanked for her suggestion, which shall be considered. She is of opinion that nurses who subscribe to procure a pension at a given age without the return of the premiums, should be treated as are participants in the Bengal Military Orphan Fund, which provides a pension of £40 a-year for officers' daughters until marriage, when this pension ceases; but a final payment of £40 is made by the fund to the bride to provide her with a trousseau.

**Nurses over Forty Years of Age.** The lady superintendent of the Establishment for Invalid Gentlewomen and Nurse Hayden, are informed that their letters shall be brought under the notice of the committee. The tables have been arranged so as to provide for the admission of nurses up to the age of fifty, who desire to join the Fund. It is feared, however, that the necessary annual contribution will be found to be larger than nurses of this age may be able to pay. Where the nurse has saved a portion of her wages, and so accumulated a lump sum, she will be able to pay it in to the Fund, if she so desires, and thus to lessen her annual contribution. All nurses who have saved money, and desire to secure a pension, are invited to send a statement of their age, the amount they have saved, and the amount per annum they think they would be able to contribute, so that each case may be specially considered.

**Cottage Hospital Nurses.** The matron of the Boston Cottage Hospital is thanked for her letter. It is the opinion of the more experienced hospital managers that a trained nurse should be paid at least £25 per annum, with board, uniform, and washing. It is felt that each nurse so circumstanced should be able to pay one-eighth of her salary as a minimum annual contribution to the Fund. The cases of widows supporting and educating children who may be absolutely dependent on them, are, we hope, exceptional, and must be dealt with as they arise.

**What a Nurse Can Pay.** Mr. P. Wigram, of the East London Nursing Society, supplies some useful information on this point. He writes: "There is no pension fund, but a lady connected with the society is ready to take care of the nurses' savings, if they choose, to invest them in the Post Office, and eleven out of twenty do so." The nurses' pay is £36 8*s.* for the first three years, and then £44 4*s.* They also get free lodgings (but not board) and uniform (cloaks, bonnets, and upper dress). Of six on £44 4*s.*, two put by about £6 yearly, two £3, and two £2 8*s.* Of five on £36 8*s.*, three put by £3, and two £2 8*s.* One of the last stopped saving for some time after her first year. Now she has risen to the higher grade, and has begun to invest again, and is doing so at the rate of £3.

This will rather show what they do put by than what they can. The best investors are the oldest, and sometimes those with heavy family drains. One who puts by £6 did so when she had three children to support, and one who saves £3 has many calls on her from her relations; indeed she says that she invests it in this way in self defence, so that she may tell them that she has no money left when they come to her.

**Nurses of Sixty and Upwards.** Mrs. Walker is referred to the answers given under the heading of "Nurses over Forty Years of Age." If she will supply the information there asked for she will be advised by the Actuary as to the best way of investing her savings so as to secure an annuity for the rest of her life.

